# Subcutaneous Adipose Tissue Accumulation Is an Independent Risk Factor of Urinary Stone in Young People

**DOI:** 10.3389/fendo.2022.865930

**Published:** 2022-06-30

**Authors:** Zixing Ye, He Xiao, Guanghua Liu, Yi Qiao, Yi Zhao, Zhigang Ji, Xiaohong Fan, Rongrong Li, Ou Wang

**Affiliations:** ^1^ Department of Urology, Peking Union Medical College Hospital, Beijing, China; ^2^ Department of Nephrology, Peking Union Medical College Hospital, Beijing, China; ^3^ Department of Clinical Nutrition, Peking Union Medical College Hospital, Beijing, China; ^4^ Department of Endocrinology, Peking Union Medical College Hospital, Beijing, China

**Keywords:** subcutaneous adipose tissue, metabolic disease, fat distribution, urinary stone, stone episode

## Abstract

**Background:**

Urinary stones usually start at a young age and tend to recur. Therefore, preventing stone occurrence and recurrence in young people is crucial. We aimed to investigate the association between subcutaneous adipose tissue, visceral adipose tissue, and stone episodes in young people.

**Methods:**

We retrospectively studied patients aged below 40 years with kidney or ureteral stones. Data on demographic and metabolic characteristics, urolithiasis history, subcutaneous fat area (SFA), and visceral fat area (VFA) were collected. We evaluated the association between SFA or VFA and the occurrence or recurrence of stone episodes using binary logistic regression and Poisson regression analyses.

**Results:**

In total, 120 patients were included. Abdominal obesity, overweight or obesity, dyslipidemia, metabolic syndrome, SFA, and VFA increased with the number of stone episodes (all p < 0.05). The increase in SFA was independently associated with episode occurrence (p = 0.015). Patients with an SFA > 97 cm^2^ had a higher risk of episode occurrence. SFA and VFA accumulation were independently associated with episode recurrence (all p < 0.05), and SFA had a stronger association than VFA did.

**Conclusions:**

In young people, SFA accumulation is an independent and early risk factor for the occurrence and recurrence of stone episodes. Subcutaneous fat could be a convenient and effective indicator to assess the risk of stone episodes before the development of metabolic disorders.

## Introduction

Urolithiasis is one of the diseases that often affect young people. Among people under 40 years of age, nearly 1 in 10 suffers from urolithiasis (1, 2). Young people are the main workforce in society, and an early onset of stones may affect their work and personal lives constantly. The prevention of stone occurrence in young people is also more cost-effective than management of stones in elderly people. Therefore, it is important to study and reduce the risk factors of urinary stones in young people. Many studies have shown the relationship between urolithiasis and metabolic disorders, especially metabolic syndrome (MetS) (3–5). Therefore, the earlier the metabolic disorder is controlled, the more likely it is to prevent urinary stones. However, most of the metabolic disorders that are found to be related to urinary stones are the result of a long-term decompensation of the metabolic system. Few studies have investigated stone formation in young people. The accumulation of subcutaneous adipose tissue (SAT) occurs when the metabolic imbalance is compensable. Some studies on Asian and African populations have shown a relationship between SAT and MetS (6–8). However, current studies seem to show that visceral adipose tissue (VAT) is more closely related than SAT to stone formation in patients of all ages. Most current studies have investigated the effect of metabolic disorders on the urinary component instead of the stone episodes that really affected young people’s quality of life and work.

In this study, we aimed to investigate the association between SAT, VAT, and urinary stone episodes, with the intention of identifying the risk factors of the stone episodes, and then provide effective methods and a theoretical basis for stone prevention among young individuals.

## Materials and Methods

### Patients

This was a retrospective study involving patients from PRE-STONE, a metabolic evaluation database established by Peking Union Medical College Hospital. The inclusion criteria for the patients were as follows: the patients had to be (1) aged 18 years or over but below 40 years; (2) diagnosed with kidney or ureteral stone by either a definite kidney or ureteral stone history or abdominopelvic non-contrast computed tomography (NCCT); and (3) have completed metabolic evaluation in our hospital between May 2016 and January 2019. The exclusion criteria were as follows: (1) patients on lipid-lowering medications; (2) patients taking stone-related medications such as potassium citrate, allopurinol, or thiazides; (3) patients with a known metabolic or anatomic cause of urolithiasis; or (4) patients with a history of bariatric surgery or surgery in the upper urinary tract. A stone episode was defined as the onset of renal colic, hematuria, hydronephrosis, UTI, or flank pain related to urinary stones. The occurrence of stone episodes was defined as the presence of at least one stone episode. Recurrence of stone episodes was defined as the occurrence of at least two episodes. Only stone episodes that occurred in the past 10 years were considered for the purposes of this study.

### Data Collection

Demographic and clinical data, including age, gender, history of stone episodes, family history of kidney or ureteral stone, past medical history, medications, vital signs, laboratory blood tests, and imaging examinations were recorded. Two urologists, both with three years of experience in the metabolic evaluation of urinary stones, collected the demographic and clinical data, and evaluated the history of SF with regard to stone episodes by face-to-face consultation. The results of blood tests were collected from clinical laboratory, and imaging examinations were collected from Picture Archiving and Communication Systems (PACS).

### Diagnostic Criteria

The definition of MetS proposed by International Diabetes Federation in 2005 (9) was adopted. In this study, a body mass index (BMI) of 28 kg/m^2^ or more was considered obesity and a BMI of 24 kg/m^2^ or more, but less than 28 kg/m^2^, was considered overweight (10). Abdominal obesity was defined as a waist circumference of 90 cm or more in males and 80 cm or more in females (11). Diabetes mellitus (DM) was considered if the patient had a fasting blood glucose level of 7.0 mmol/L or more, or a history of DM, or was taking antidiabetic medications (12). Dyslipidemia was defined as the occurrence of one of the following disorders: hypercholesterolemia (>5.17 mmol/L), hypertriglyceridemia (≥ 1.7 mmol/L), low levels of high-density lipoprotein cholesterol (<1.04 mmol/L for males and <1.30 mmol/L for females), or high levels of low-density lipoprotein cholesterol (>3.36 mmol/L) (13). Renal insufficiency was considered with estimated glomerular filtration rate (eGFR) <90 mL/min (14). The value of eGFR was calculated using the Modification of Diet in Renal Disease (MDRD) Study equation (15).

### Visceral and Subcutaneous Adipose Tissue Assessment

Visceral fat area (VFA) and subcutaneous fat area (SFA) were measured at the umbilical level on an NCCT axial slice and used as an indicator to estimate the total amount of abdominal VAT and SAT. The adipose tissue area was delineated by two urologists using ImageJ software (http://rsb.info.nih.gov/ij/). The VFA and SFA were measured as pixels within an attenuation range of −250 to −50 Hounsfield units, also using the ImageJ software (16). The data of SFA and VFA were converted to one tenth of the original value (10 cm^2^). All NCCT examinations were performed using a third-generation dual-source computed tomographic (CT) system (Somatom Force, Siemens Healthcare, Forchheim, Germany) with a regular dose of 120 kV and a slice thickness of 5 mm. The scan was performed by technicians in the Department of Radiology of our hospital.

### Statistical Analysis

Demographic and clinical data were expressed as means ± standard deviation for continuous variables with a normal distribution and as percentage for categorical variables. Between-group comparisons for categorical variables were performed using chi-squared test. One-way analysis of variance (ANOVA) was used for evaluation of continuous variables with a normal distribution, and non-parametric analysis was performed for continuous variables with an abnormal distribution. The association between the occurrence or recurrence of stone episode and SFA or VFA was calculated using the binary logistic regression model (age, gender, family history of stone, MetS, and renal insufficiency were included as covariates). The association between SFA or VFA and the number of stone episodes was assessed using Poisson regression. Covariates in Poisson regression included age, gender, family history of stone, MetS, and renal insufficiency. A receiver-operating characteristic (ROC) curve analysis was used to evaluate the effects of SFA and VFA in estimating the occurrence of stone episode. The area under the curve (AUC) was used to evaluate the performance of variables in identifying stone episodes. All tests were of two-tailed type, and a *p* value of <0.05 was considered statistically significant. Statistical analysis was performed using IBM SPSS Statistics for Windows, version 23.0 (IBM Corp., Armonk, NY, USA).

## Results

### Demographic and Metabolic Characteristics

Based on the inclusion criteria, 131 patients were included in this study. Of these, 11 were excluded, including nine patients who had a known metabolic or anatomic cause of urolithiasis (five patients with polycystic kidney, two with medullary sponge kidney, one with calyceal diverticula, and one with renal tubular acidosis), one patient who was using the lipid-lowering medication atorvastatin, and one who was using stone-related medication potassium citrate. Therefore, a total of 120 patients, including 86 (71.7%) men and 34 (28.3%) women, were enrolled in this study. The patients had an average age of 31.9 ± 5.2 years. We observed that the proportion of abdominal obesity, overweight or obesity, dyslipidemia, and MetS, as well as the values of SFA and VFA, increased significantly with the increase in the number of stone episodes (p < 0.05). However, there were no statistical differences between stone episodes and family history, hypertension, DM, or renal insufficiency (p > 0.05; **Table 1**).

**Table 1:** Demographic and metabolic characteristics of SFs with different stone episodes.

Characteristics	All patients	Number of stone episodes			
		Asymptomatic	Once	Twice or more	*p*
Number of patients	120	48	38	34	
Gender (male/female; male [%])	86/34 [71.7]	29/19 [60.4]	29/9 [76.3]	28/6 [82.4]	0.025
Age (year)	31.9 ± 5.2^*^	30.40 ± 5.26	32.66 ± 5.25	33.1 ± 4.6	0.037
Family history (n [%])	50 [41.7]	21 [43.8]	27 [71.1]	18 [52.9]	0.111
BMI (kg/m^2^)	25.4 ± 4.0	24.0 ± 3.9	24.9 ± 3.1	27.7 ± 4.1	< 0.001
Abdominal obesity (n [%])	53 [44.2]	12 [25.0]	17 [44.7]	24 [70.6]	< 0.001
Overweight or obesity (n [%])	79 [65.8]	25 [52.1]	24 [63.2]	30 [88.2]	0.003
Hypertension (n [%])	30 [25.0]	8 [16.7]	11 [28.9]	11 [32.4]	0.215
DM (n [%])	6 [5.8]	0 [0.0]	2 [5.6]	4 [11.8]	0.087
Dyslipidemia (n [%])	80 [66.7]	23 [47.9]	27 [71.1]	30 [88.2]	0.001
MetS (n [%])	35 [29.2]	4 [8.3]	12 [31.6]	19 [55.9]	< 0.001
Renal insufficiency (n [%])	25 [23.1]	9 [23.7]	7 [28.0]	9 [26.5]	0.707
VFA (10 cm^2^)	19.2 ± 9.6	17.1 ± 7.7	16.6 ± 7.1	25.0 ± 11.8	0.001
SFA (10 cm^2^)	10.5 ± 5.9	8.0 ± 5.1	10.7 ± 5.4	13.9 ± 6.0	< 0.001

^*^Mean ± SD (all such value).

Continuous variables with a normal distribution were expressed as means ± standard deviation, categorical variables were expressed as percentage. Chi-squared test was used for between-group comparisons of categorical variables. One-way analysis of variance (ANOVA) was used for evaluation of continuous variables with a normal distribution, and non-parametric analysis was used for continuous variables with an abnormal distribution. The p value was considered significant if less than 0.05.

DM, diabetes mellitus; MetS, metabolic syndrome; SF, stone former; SFA, subcutaneous fat area; VFA, visceral fat area.

### SFA Accumulation as an Independent Risk Factor of Stone Episode Occurrence and Recurrence

In all SFs, the increase in SFA was significantly associated with the occurrence of stone episodes (OR, 1.150; 95% CI, 1.028–1.286; p = 0.015) after adjustment. These factors, including MetS (OR, 3.271; 95% CI, 0.858–12.463; p = 0.083), were not related to the occurrence of stone episodes. Even in SFs without MetS, the increase in SFA was still significantly associated to the occurrence of stone episodes after adjustment (OR, 1.131; 95% CI, 1.006–1.271; p = 0.039). No significant association was found between VFA and the occurrence of stone episodes (p > 0.05; **Table 2**).

**Table 2:** The relationship between SFA, VFA, and the occurrence of stone episode.

Variables	All SFs				SFs without MetS			
	Unadjusted		Adjusted^¶^		Unadjusted		Adjusted^§^	
	OR (95%CI)	*p*	OR (95%CI)	*p*	OR (95%CI)	*p*	OR (95%CI)	*p*
SFA	1.159 (1.070–1.254)	< 0.001	1.150 (1.028–1.286)	0.015	1.092 (1.013–5.691)	0.047	1.131 (1.006–1.271)	0.039
VFA	1.043 (0.999–1.090)	0.058	1.014 (0.954–1.077)	0.658	0.985 (0.929–1.044)	0.614	0.991 (0.922–1.066)	0.813

^¶^Adjusted for age, gender, family history of stone, MetS, and renal insufficiency.

^§^Adjusted for age, gender, family history of stone, and renal insufficiency.

The binary logistic regression model was used to assess the association between the occurrence of stone episode and SFA or VFA.

OR, odds ratio; CI, confidence interval; MetS, metabolic syndrome; SF, stone former.

SFA and VFA were all associated with the recurrence of stone episodes, and SFA has a stronger association (OR, 1.145; 95% CI, 1.021–1.284; p = 0.021) than VFA (OR, 1.113; 95% CI, 1.035–1.197; p = 0.004) after adjustment. In SFs without MetS, SFA is still associated with the recurrence of stone episode (OR, 1.115; 95% CI, 1.004–1.239; p = 0.042), although the relationship was not statistically significant after the adjustment (p > 0.05; **Table 3**).

**Table 3:** The relationship between SFA, VFA, and the recurrence of stone episode.

Variables	All SFs				SFs without MetS			
	Unadjusted		Adjusted^¶^		Unadjusted		Adjusted^§^	
	OR (95%CI)	*p*	OR (95%CI)	*p*	OR (95%CI)	*p*	OR (95%CI)	*p*
SFA	1.162 (1.069–1.262)	< 0.001	1.145 (1.021–1.284)	0.021	1.115 (1.004–1.239)	0.042	1.142 (0.981–1.328)	0.086
VFA	1.100 (1.045–1.156)	< 0.001	1.113 (1.035–1.197)	0.004	1.052 (0.981–1.129)	0.155	1.075 (0.977–1.183)	0.137

^¶^Adjusted for age, gender, family history of stone, MetS, and renal insufficiency.

^§^Adjusted for age, gender, family history of stone, and renal insufficiency.

The binary logistic regression model was used to assess the association between the recurrence of stone episode and SFA or VFA.

OR, Odds ratio; CI, Confidence interval; MetS, metabolic syndrome; SF, stone former.

The Poisson regression in **Table 4** showed a significant association between SFA and number of stone episodes in all SFs (beta, 1.037; 95% CI, 1.000–1.076; p = 0.049). The number of stone episodes increased by 3.7% on average for every 10 cm^2^ increase in the SFA (p = 0.049) after adjustment. The association between SFA and the number of stone episodes was also significant before adjustment (beta, 1.054; 95% CI, 1.006–1.104; p = 0.026) and less statistically significant after adjustment (beta, 1.058; 95% CI, 0.998–1.121; p = 0.060) in SFs without MetS. However, no significant association was observed between VFA and the number of stone episodes (p > 0.05).

**Table 4:** The relationship between SFA, VFA, and the number of stone episodes in all SFs and those without MetS.

Variables	All SFs				SFs without MetS			
	Unadjusted		Adjusted^¶^		Unadjusted		Adjusted^§^	
	Exp(β) (95% CI)	*p*	Exp(β) (95% CI)	*p*	Exp(β) (95% CI)	*p*	Exp(β) (95% CI)	*p*
SFA	1.056 (1.027–1.085)	< 0.001	1.037 (1.000–1.076)	0.049	1.054 (1.006–1.104)	0.026	1.058 (0.998–1.121)	0.060
VFA	1.026 (1.009–1.044)	0.002	1.018 (0.997–1.039)	0.100	1.007 (0.972–1.042)	0.709	1.011 (0.971–1.051)	0.599

^¶^Adjusted for age, gender, family history of kidney stone, MetS, and renal insufficiency.

^§^Adjusted for age, gender, family history of kidney stone, and renal insufficiency.

Poisson regression was used to evaluate the association between the number of stone episodes and SFA or VFA.

CI, confidence interval; MetS, metabolic syndrome; SF, stone former.

### SFA as an Indicator to Identify the Occurrence of Stone Episodes

**Figure 1:**
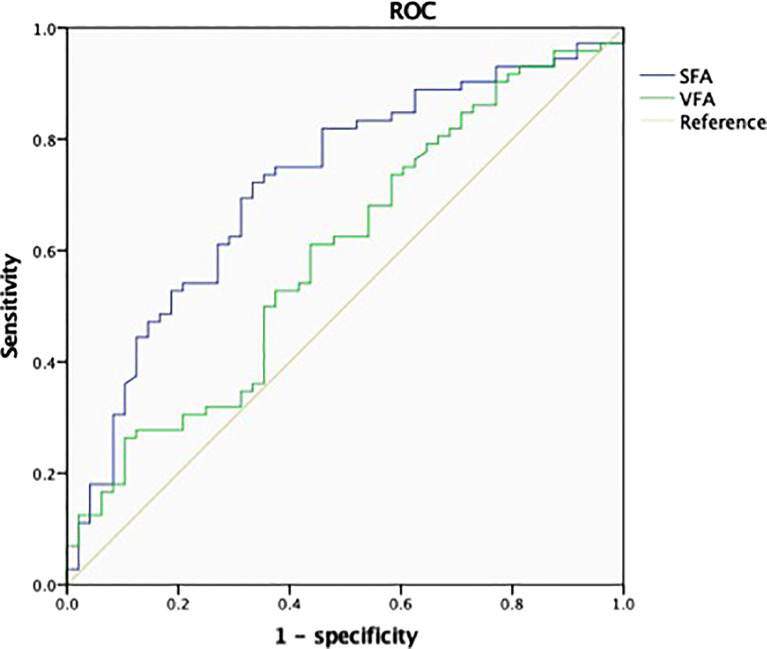
Receiver operating characteristic (ROC) analysis and area under the curve (AUC) for the predictors of the occurrence of stone episode. The AUC values of subcutaneous fat area (SFA) and (visceral fat area) VFA were 0.718 and 0.594 with 95% confidence interval (CI) of 0.624–0.812 and 0.490–0.698, respectively. The cutoff point of SFA was 97 cm^2^ and that of VFA was 174 cm^2^. ROC analysis was performed for the occurrence of stone episode based on sensitivity of 72.2%, specificity of 66.7%, and the Youden index of 0.389 for SFA and sensitivity of 61.1%, specificity of 56.2%, and the Youden index of 0.173 for VFA.

In this study, ROC curves were plotted to compare between VFA and SFA in terms of the predictive power for the occurrence of the stone episode. The ROC curve analysis with the AUC of VFA and SFA are demonstrated in **Figure 1**. The AUC of SFA (AUC_SFA_ = 0.718; 95% CI, 0.624–0.812) was significantly more than the AUC of VFA (AUC_VFA_ = 0.594; 95% CI, 0.490–0.698), and SFA was adopted as an indicator of the stone episode occurrence. The cutoff point of SFA for predicting the occurrence of stone episode was 97 cm^2^ in all SFs based on ROC curve analysis, with sensitivity of 72.2%, specificity of 66.7%, and the Youden index of 0.389; while the cutoff point of VFA was 174 cm^2^, with a sensitivity of 61.1%, specificity of 56.2%, and a Youden index of 0.173.

## Discussion

Many studies have shown the association between urolithiasis and MetS or its components (3–5). VAT seems to have a closer relationship with urinary stone than SAT does among people of all ages (17–19). SAT is insulin-sensitive and is considered an “energy sink” to store excess energy physiologically, the visceral fat begins to increase when the subcutaneous adipose tissue is unable to handle the calorie surplus due to excess energy accumulation (6).Thus, the accumulation of SAT should happen earlier than the increase in VAT and MetS. Theoretically, it should be more effective to reduce the risk of stone episodes by reducing the SAT when the SFs are young. However, few studies have reported the association between SAT and stone episodes among young people.

Metabolic disorders are associated with stone episodes. Demographic results in this study showed that the prevalence of abdominal obesity, overweight or obesity, dyslipidemia, and MetS significantly increased with the number of stone episodes in young SFs (*p* < 0.05). This is consistent with the current understanding that metabolic disorders are associated with urolithiasis in SFs of all ages (3, 20). However, there are still some controversies on the effect of hypertension and DM on urolithiasis (21). A prospective study showed that hypertensive patients tended to have a higher risk of stone formation; however, SFs in this hypertensive group also had a higher unadjusted BMI, which was also the risk factor of urolithiasis (22). The prospective studies performed by Madore also showed that kidney stones preceded the development of hypertension (23, 24). In our study, the proportion of DM and hypertension increased with stone episodes; however, statistical significance was not achieved. This result may partly be attributed to the fact that urolithiasis and hypertension may share some risk factors, and a larger sample size is needed in further studies.

Accumulation of SAT was independently related to the occurrence of stone episodes in young SFs. In our study, we found that SFA was related to the occurrence of stone episodes independent of MetS in young SFs. This was consistent with the fact that SAT accumulation occurs earlier than MetS (6). Previous studies did not show the relationship between SFA and episode occurrence in SFs of all ages. This may be because as the SAT increases with age, the difference of SAT reduces among elder SFs. Therefore, it would be more effective to reduce SAT accumulation than to treat MetS to prevent the occurrence of stone episodes. Furthermore, the ROC curve showed that SFA is superior to VFA as an indicator of the occurrence of stone episodes in young SFs. Only one cutoff value of VFA had been proposed prior to our study to evaluate the risk of urolithiasis, and VFA categorization was not based on the predictive efficacy of VFA on real-world stone episodes or 24-h urinary abnormalities (25). Based on our results of the AUC analysis, we proposed the first cutoff value of SFA to predict the occurrence of stone episode, and suggested that SFs with an SFA of >97 cm^2^ were more likely to experience stone episodes. The clinical value of our results is that young SFs may be able to prevent stone episodes that may affect their quality of life by reducing SFA.

Fujimura et al. found that visceral fat accumulation was significantly related to stone formation in male patients (95% CI, 1.009–1.021; p < 0.001) (17). Some studies also showed that VFA was related to abnormal urine components. Fram et al. suggested that VFA was significantly associated with an increased excretion of urine sodium in male patients with either normal or increased BMI (19). However, our study showed no significant association between the VFA and the occurrence of stone episodes. This may be explained by the fact that the accumulation of VAT occurs later than that of SAT and the increasing of VAT is generally not so evident among young SFs. Moreover, the patients’ work and quality of life were not affected by abnormal urine components. Few studies have studied the effect of VAT on stone episodes in young SFs. Further investigation may be required to determine the clinical significance of VAT in young SFs.

Both SFA and VFA were independently related to the recurrence of stone episodes, and SFA had a closer relationship with stone recurrence than VFA did in our study. Yamashita and Bos both found that recurrent SFs had a higher %VFA (%VFA=VFA/[VFA + SFA]) than first-time SFs of all ages (16, 26). Several studies have shown a correlation between metabolic disorders and stone recurrence as well (27, 28). However, the SFs in above-mentioned studies were in their fifties, much older than SFs in our study. The accumulation of SAT begins earlier than VAT, thus the difference in SFA between SFs with and without recurrence of stones tends to be more significant than the difference in VFA. Similarly, we found that the SFA was associated with the number of stone episodes in all SFs, which increased at an average of 3.7% per 10 cm^2^ of SFA. This suggested that a slight increment in the SFA might increase the risk of stone episodes, which highlights the significance of controlling the SAT in young SFs. Although the association between SFA and the number of stone episodes was less significant after adjustment, this might due to the small sample size.

Race may also be an important factor for the closer relationship of the occurrence and recurrence of stone episodes with SFA. Previous studies have shown that VFA or %VFA were associated with stone formation or recurrence; however, most of these studies were performed in Caucasian populations (16, 19). Some studies showed that SAT was related to MetS in Asians and Africans (7, 8). Therefore, more studies are needed to verify the relationship between SAT and urinary stones in both young and elderly Chinese individuals.

We found that SFA was a better independent indicator than VFA in assessing the risk of occurrence and recurrence of stone episodes and the number of stone episodes in young SFs. Based on this finding, young SFs could assess their risk of occurrence and recurrence of stone episodes simply by measuring their abdominal subcutaneous fat in a quick test. This will enable young SFs to take actions to prevent future stone episodes as early as the compensation state of metabolic imbalance, instead of waiting until MetS develops.

Our study has a few limitations. First, it was a single-center retrospective study, and all subjects enrolled in this study were SFs. Patients who did not or were unwilling to complete the metabolic evaluation were not included. Second, the number of patients enrolled in this study was relatively small; therefore, a larger sample size is needed to verify the results.

## Conclusion

The accumulation of SFA is an independent risk factor for the occurrence and recurrence of stone episodes in young people. Self-measurement of subcutaneous fat may be a very simple and effective method to assess the risk of stone episodes and could help prevent their occurrence or recurrence even before the development of metabolic disorders. The association between stone episodes and SFA may also help further explore the mechanism of urolithiasis.

## Data Availability Statement

The raw data supporting the conclusions of this article will be made available by the authors, without undue reservation.

## Ethics Statement

This study was reviewed and approved by the Institutional Review Board of Peking Union Medical College Hospital (Protocol number: S-K1961). All data was collected after obtaining signed informed consent of the patients.

## Author Contributions

Study design: ZY, YQ, and HX; data collection: YZ, HX, GL, YQ, YZ, ZJ, XF, RL, and OW; data analysis: ZY, HX, and GL; statistical analysis and data interpretation: ZY and YZ; literature search: ZY, HX, GL, YQ, YZ, XF, RL, and OW; generation of figures: ZY and HX; writing of the manuscript: ZY and HX. All authors had final approval of the submitted and published versions.

## Funding

This research was supported by The National Key Research and Development Program of China (2021YFC2009300, 2021YFC2009306).

## Conflict of Interest

The authors declare that the research was conducted in the absence of any commercial or financial relationships that could be construed as a potential conflict of interest.

## Publisher’s Note

All claims expressed in this article are solely those of the authors and do not necessarily represent those of their affiliated organizations, or those of the publisher, the editors and the reviewers. Any product that may be evaluated in this article, or claim that may be made by its manufacturer, is not guaranteed or endorsed by the publisher.
